# Induced pemphigus erythematosus after treatment for plaque psoriasis with secukinumab: a case report

**DOI:** 10.3389/fimmu.2025.1612422

**Published:** 2025-09-10

**Authors:** Fengming Hu, Hong Peng, Jian Gong, Xiaohua Tao, Xi Wang, Jianwei Bao, Pingxiu He, Lismin Dirwanto, Mingqiang Liu, Hongbing Yang

**Affiliations:** ^1^ Clinical Medical College, Jiangxi University of Chinese Medicine, Nanchang, China; ^2^ Dermatology Hospital of Jiangxi Province, Jiangxi Provincial Clinical Research Center for Skin Diseases, National Clinical Research Center for Skin Diseases, The Affiliated Dermatology Hospital of Nanchang University, Nanchang, China; ^3^ International Education College, Jiangxi University of Chinese Medicine, Nanchang, China

**Keywords:** pemphigus erythematosus, secukinumab, adverse drug event, psoriasis, autoimmune disease

## Abstract

Secukinumab is a fully human monoclonal antibody that specifically targets and neutralizes interleukin (IL)-17A, a cytokine typically involved in the mucocutaneous defense against pathogens. Despite its favorable safety profile, serious adverse events such as inflammatory bowel disease, oral pemphigoid-like lesions, and lupus erythematosus have been reported. While widely used for psoriasis treatment, cytokine-based therapies carry a potential risk of triggering autoimmune responses. The precise mechanism by which secukinumab induces pemphigus erythematosus remains unclear but may involve immune dysregulation, autoantibody production, microbiota influences, and genetic susceptibility. We report a case of pemphigus erythematosus occurring in a psoriasis patient during secukinumab therapy, which improved after treatment with methylprednisolone sodium succinate injection, leading to clinical symptom resolution. Clinicians should remain vigilant for potential complications and develop individualized management strategies for patients receiving secukinumab.

## 1 Introduction

Pemphigus encompasses a group of chronic blistering diseases affecting mucous membranes and skin, including pemphigus vulgaris, pemphigus vegetans, pemphigus foliaceus, and pemphigus erythematosus, along with variants such as paraneoplastic pemphigus, IgA pemphigus, drug-induced pemphigus (DIP), and pemphigus herpetiformis. Pemphigus is triggered by IgG autoantibodies targeting keratinocyte adhesion proteins (desmogleins Dsg1 and Dsg3). Binding of IgG autoantibodies to desmosomal complexes causes intraepithelial adhesion loss (acantholysis), resulting in skin/mucosal blisters, bullae, and erosions (1). Although pemphigus erythematosus is recognized as an autoimmune disorder, the specific mechanism of desmosome destruction following autoantibody binding remains incompletely understood. Characteristic histopathological features include superficial epidermal acantholysis leading to subcorneal blisters, dermal edema, and perivascular inflammatory infiltrates; chronic lesions may show epidermal hyperplasia and parakeratosis. Direct immunofluorescence (DIF) reveals the characteristic intercellular net-like deposition of IgG and C3 within the epidermis. Indirect immunofluorescence (IIF) detects serum-specific anti-intercellular epidermal antibodies (primarily anti-desmoglein 1 antibody), with titers correlating positively with disease activity. Notably, approximately 30%-80% of patients exhibit positive antinuclear antibodies (ANA), but concomitant cases meeting systemic lupus erythematosus diagnostic criteria remain rare (2). Herein, we report the case of a 54-year-old woman who developed pemphigus erythematosus three weeks after initiating secukinumab for psoriasis. Based on the temporal correlation between drug initiation and lesion onset, secukinumab-induced pemphigus erythematosus was suspected. While secukinumab has been reported to induce other autoimmune diseases (3), to the best of our knowledge, this represents the first documented global case of secukinumab-induced pemphigus erythematosus.

## 2 Case presentation

**Figure 1:**
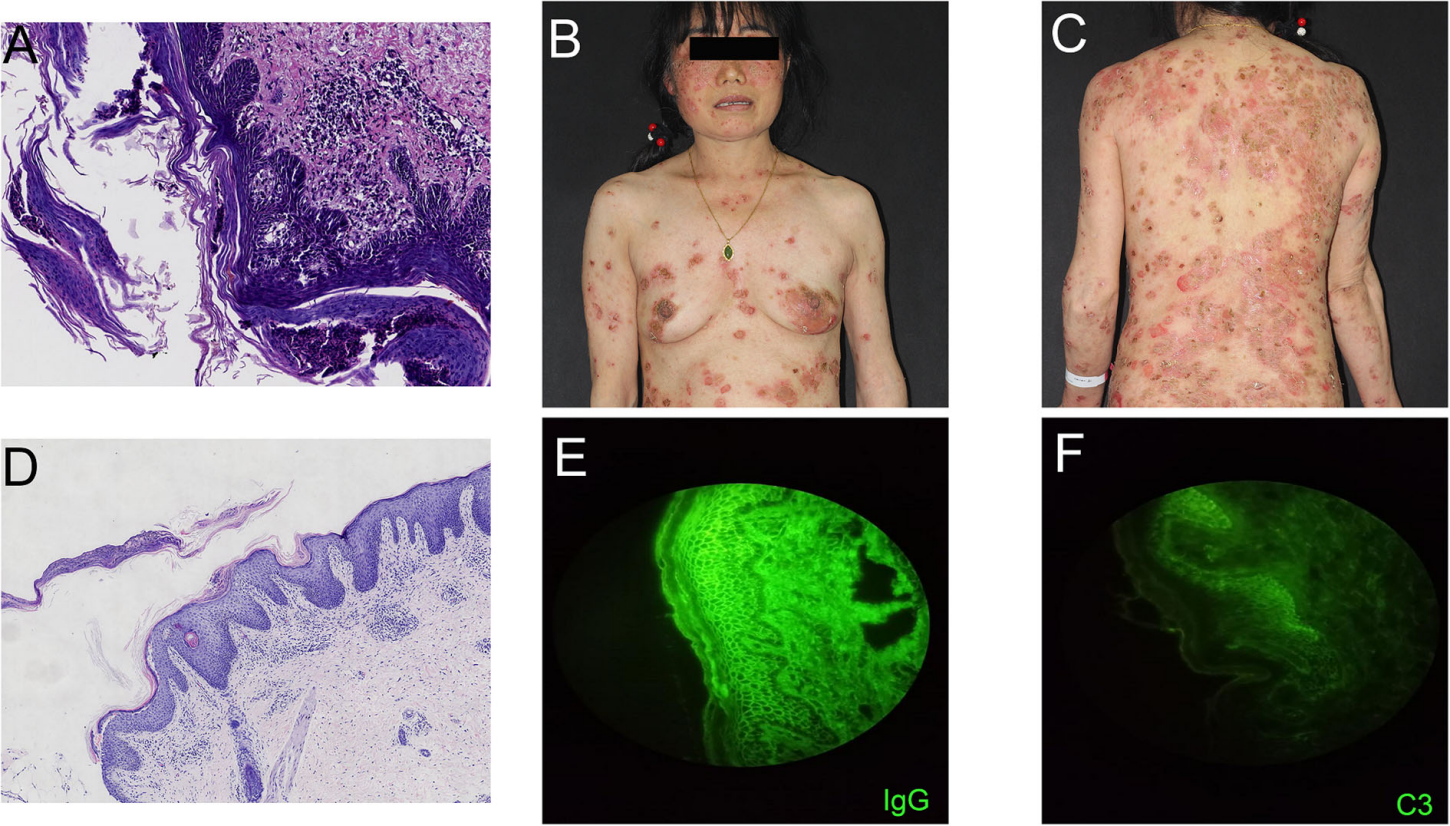
**(A)** Skin biopsy reveals extensive confluent hyperkeratotic lesions within the epidermis, accompanied by neutrophil infiltration forming Munro microabscesses; acanthocyte proliferation with partial acantholytic necrosis leading to spongiotic edema; rete ridges exhibit club-like elongation extending into the dermis. Dermal papillae project upward into the epidermis, with overlying epidermal atrophy; capillaries within the dermal papillae show proliferation, tortuosity, and dilation. Perivascular inflammatory cell infiltration is observed in the superficial dermis ((H&E; magnification, 100x). **(B, C)** Generalized bright red vesicles, thick yellow seborrheic crusts, as well as blisters and flaccid pustules on erythematous or normal skin are present. **(D)** Skin biopsy (right thigh) demonstrates hyperkeratosis, surface crusting, acantholysis in the granular layer and upper spinous layer, and intraepidermal cleft formation. Scattered perivascular inflammatory infiltrates composed predominantly of lymphocytes and eosinophils are seen in the superficial dermis ((H&E; magnification, 40x). **(E, F)** Direct immunofluorescence shows linear deposition of IgG and complement C3 at the dermoepidermal junction, while IgA and IgM are negative.

**Figure 2:**
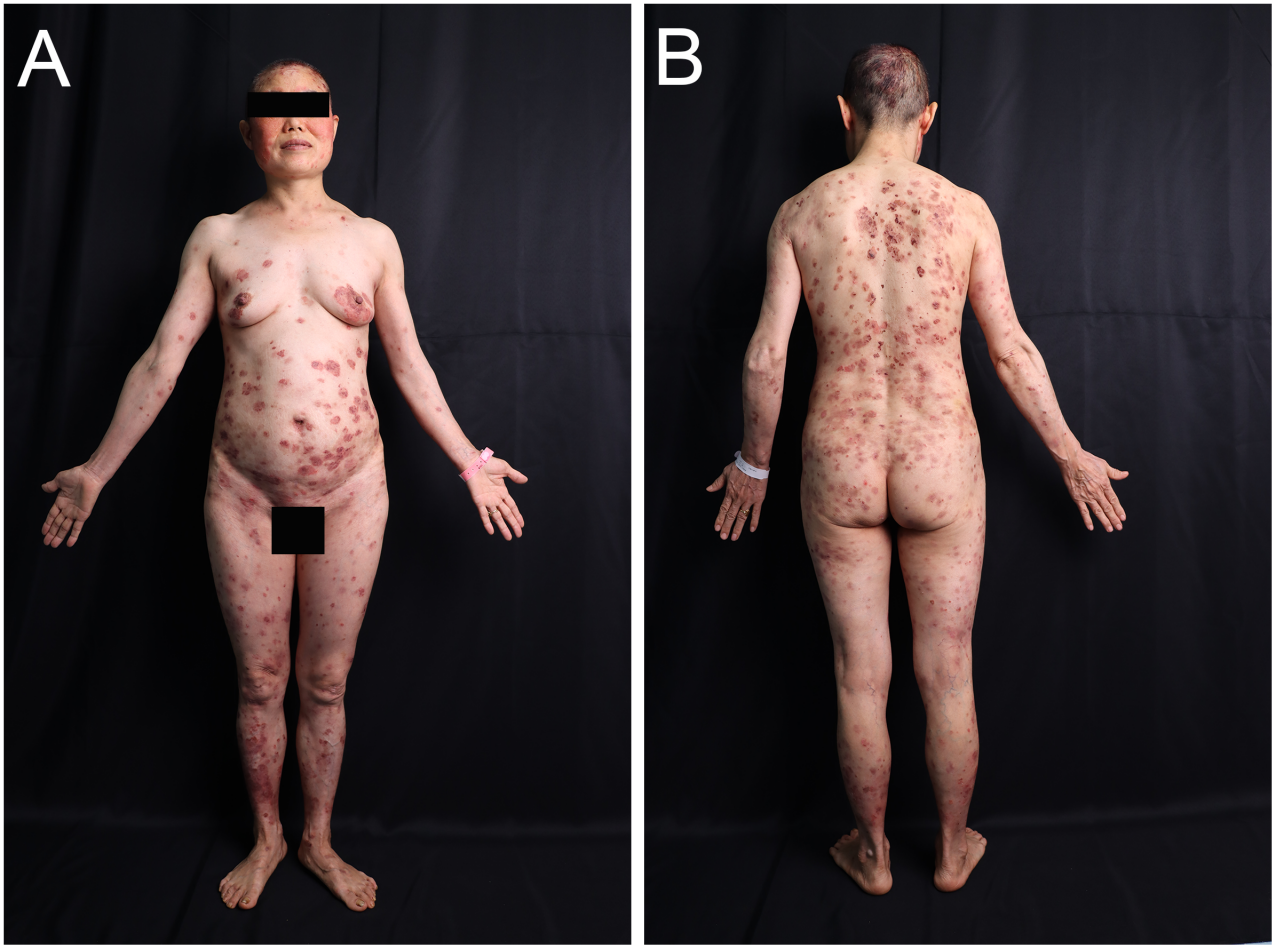
Images of the patient after treatment. **(A, B)** Significant improvement in lesions after glucocorticoid treatment.

**Figure 3:**
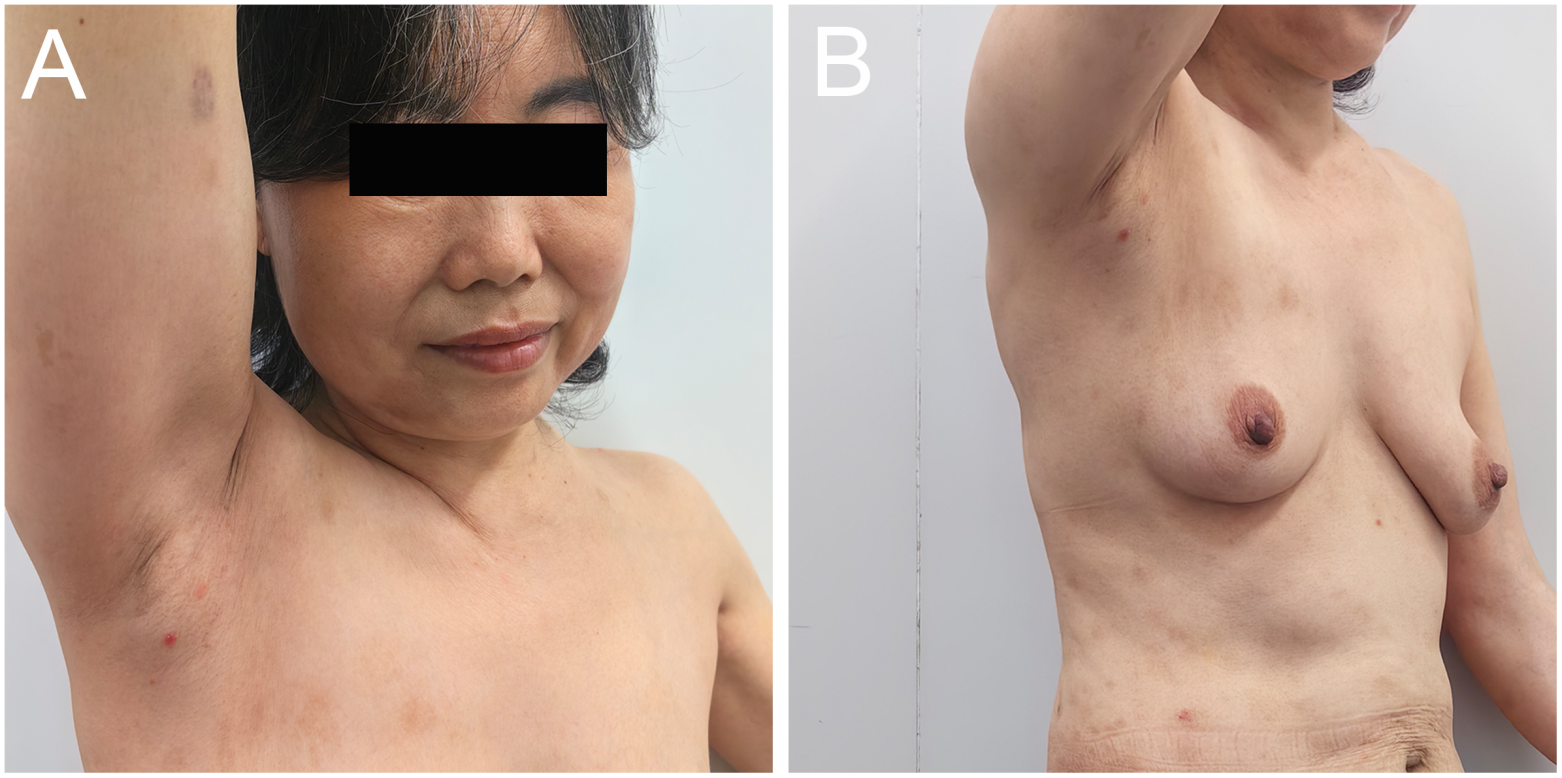
Images of the patient relapsing. **(A, B)** Recurrence of psoriatic lesions 10 months after discharge when glucocorticoid administration was terminated.

A 54-year-old woman with a 5-year history of psoriasis presented to our Department of Integrated Traditional Chinese and Western Medicine. Histopathological examination revealed: extensive confluent parakeratotic foci containing neutrophilic microabscesses (Munro microabscesses); acanthosis with partial keratinocyte acantholysis and necrosis; spongiosis; club-shaped elongation of rete ridges; papillomatosis with overlying epidermal atrophy; and dilated, tortuous capillaries within the dermal papillae, accompanied by perivascular inflammatory cell infiltration in the superficial dermis (H&E, x100), confirming psoriasis (**Figure 1A**). Previous treatment with topical corticosteroids was ineffective. Due to widespread, recalcitrant lesions and the involvement of the IL-17A pathway in the disease pathology, the patient was initiated on secukinumab 300 mg subcutaneously. Three weeks post-initiation, erythematous plaques with pruritus appeared on her face. Suspecting a psoriasis flare, secukinumab was continued for two more doses. The rash progressively generalized, evolving into erythema and flaccid blisters, some of which ruptured forming yellow-crusted erosions (**Figures 1B, C**) that were slow to heal. Physical examination revealed widespread, round or oval red macules and patches on the face, trunk, and limbs. Some erythematous bases showed bright red erosions with yellow, greasy crusts, while others or adjacent normal skin exhibited flaccid vesicles/pustules with a positive Nikolsky sign. Scattered scaly plaques were also present. Mild facial edema and white ocular discharge were noted. No oral or genital blisters, erosions, or exudate were observed. Laboratory findings included: eosinophil count 0.36x10^9/L (↑), eosinophil percentage 7.00% (↑), IgE 248.10 IU/mL (↑). Autoantibodies: anti-Dsg1 antibody 209.27 U/mL (normal <14.00), anti-Dsg3 antibody 0.82 U/mL (normal <7.00), BP180 antibody 1.40 U/mL (normal 0-9.00). ANA titer <1:100. Anti-dsDNA (IgG), anticardiolipin antibodies (IgA, IgM, IgG) were negative. Salt-split skin IIF (IgG) was negative (**Table 1**). A skin biopsy from the right thigh showed hyperkeratosis, parakeratosis, superficial crusting, acantholysis in the upper granular and spinous layers with intraepidermal cleft formation, and a superficial perivascular inflammatory infiltrate composed predominantly of lymphocytes and eosinophils (**Figures 1D**, H&E, x40). DIF of the right thigh biopsy revealed linear deposition of IgG and C3 at the dermoepidermal junction (DEJ); IgA and IgM were negative (**Figures 1E, F**). Based on these findings, a diagnosis of pemphigus erythematosus was established. Secukinumab was discontinued. Treatment with methylprednisolone sodium succinate (32 mg IV daily) led to significant improvement, with resolution of psoriasis lesions observed during this period (**Figures 2A, B**). During follow-up, the patient was maintained on low-dose oral corticosteroids without new lesions. However, 10 months after discharge, she developed pea-sized red papules with silvery-white scales on her right axilla and lower abdomen (**Figures 3A, B**).

**Table 1:** Laboratory test report.

Test	Result	Reference Range	Unit	Status
**Eosinophil Count**	0.36	0.02–0.50	×10^9^/L	Normal
**Eosinophil Percentage**	**7.00 ↑**	0.4–6.0	%	**Elevated**
**Total IgE**	**248.10 ↑**	0–100	IU/mL	**Elevated**
**Anti-Desmoglein 1 (Dsg1)**	**209.27 ↑**	<14.00 (Negative)	U/mL	**Strongly Elevated**
Anti-Desmoglein 3 (Dsg3)	0.82	<7.00 (Negative)	U/mL	Normal
BP180 Antibody	1.40	0–9.00 (Negative)	U/mL	Normal
**ANA Titer**	<1:100	Negative: <1:100	Titer	Negative
Anti-dsDNA Antibody (IgG)	Negative	Negative	-	Negative
Anti-Cardiolipin IgA	Negative	Negative	-	Negative
Anti-Cardiolipin IgM	Negative	Negative	-	Negative
Anti-Cardiolipin IgG	Negative	Negative	-	Negative
**Salt-Split Indirect IF (IgG)**	Negative	Negative	-	Negative

Bold: Indicates abnormalities or findings that require special attention.

## 3 Discussion

Pemphigus erythematosus, also known as Senear-Usher syndrome, is considered a subtype of pemphigus foliaceus. It predominantly affects elderly women, exhibiting overlapping clinical and immunological features of pemphigus foliaceus and cutaneous lupus erythematosus (4, 5). Lesions favor the seborrheic areas of the face, scalp, chest, and back, sometimes extending to the axillae and groin, but rarely involve the extremities. It primarily presents with erythema and scaling, requiring differentiation from lupus erythematosus and seborrheic dermatitis. The exact pathogenesis is unknown but may involve shared genetic markers and pathways regulating CD4+ T cells, such as IRF8 and STAT1 expression (6). Sun exposure has also been reported to exacerbate the condition (7). Senear and Usher proposed a potential link between pemphigus and lupus, possibly related to “shared autoimmunity,” with early mechanistic research focusing on the molecular level (8).

Drugs inducing DIP are categorized into thiol-containing drugs (e.g., penicillamine, captopril, bucillamine), phenol-containing drugs (e.g., aspirin, rifampicin, levodopa), and non-thiol/non-phenol drugs. Thiol drugs activate proteases and interfere with desmoglein function. Phenol drugs induce acantholysis via complement and protease modulation. Secukinumab, a non-thiol/non-phenol drug, may induce DIP through various mechanisms, such as activating autoantibodies or altering target antigen structures on keratinocytes (9). Thiol drugs typically induce pemphigus foliaceus, while non-thiol drugs often cause pemphigus vulgaris (10, 11). This case presented as pemphigus erythematosus, a phenotype not previously reported in the literature.

Reviewing prior literature, common adverse events associated with secukinumab include inflammatory bowel disease, eczematous drug eruptions, drug-associated vasculitis, and drug-induced lupus (12). Psoriasis is primarily driven by Th1/Th17 immunity, whereas pemphigus erythematosus is characterized by Th2 predominance. It is hypothesized that blocking the Th1/Th17 pathway may induce a counter-regulatory shift towards Th2 activation (13). In this case, the marked elevation of anti-Dsg1, eosinophilia, and dermal eosinophilic infiltrate suggest that IL-17A inhibition may have downregulated the Th1/Th17 axis, relieving inhibition of Th2 cells and promoting IL-4/IL-13 elevation. The eosinophilic infiltration and elevated IgE observed support Th2 predominance, potentially driving B cells to produce anti-Dsg1 autoantibodies and trigger pemphigus erythematosus. This highlights the close association between pemphigus erythematosus and type 2 inflammation involving eosinophils. The T helper 2 (Th2) pathway plays a crucial role in pemphigus. Anti-Dsg IgG production and disease severity correlate with Th2 activity. IL-4 induces Th2 proliferation, promotes isotype switching to IgG4 and IgE, and stimulates eosinophil and mast cell proliferation/degranulation (14).

Induction of pemphigus erythematosus may also involve dysregulated microbiota-immune interactions. Studies suggest IL-17 provides anti-inflammatory protection; its inhibition reduces antimicrobial peptides (e.g., β-defensins), disrupting microbial barrier balance and potentially promoting Staphylococcus aureus colonization. Superantigens from S. aureus could then activate autoreactive T/B cells (15). This inhibition might also induce polyclonal B-lymphocyte activation and autoantibody production (16). Murine studies show IL-17RA knockout leads to skin dysbiosis (e.g., increased Staphylococci and Corynebacteria), releasing antigens and activating autoimmune responses (17). Additionally, genetic susceptibility may play a role; specific HLA alleles (e.g., HLA-DRB1*04:02) could enhance susceptibility to biologic-induced autoimmunity by influencing antigen presentation efficiency (18).

## 4 Conclusion

As treatment needs for autoimmune diseases grow, the use of biologics has expanded significantly. However, associated adverse effects are increasingly recognized. With newer agents, predicting these side effects is challenging, and their underlying pathophysiology remains elusive. This case suggests secukinumab may trigger pemphigus erythematosus via Th2 immune deviation and skin dysbiosis. Clinicians using secukinumab should promptly investigate this adverse event if flaccid blisters appear on sun-exposed areas, even if DIF findings are atypical, by combining assessment with anti-Dsg1 antibody testing. Immune dysregulation induced by IL-17A inhibition likely contributed to this case. Clinicians must be aware of this potential adverse effect and consider the need for tailored management strategies and further investigation in patients receiving secukinumab.

## Data Availability

The raw data supporting the conclusions of this article will be made available by the authors, without undue reservation.
